# The Role of Gut Microbiota and Gut–Brain Interplay in Selected Diseases of the Central Nervous System

**DOI:** 10.3390/ijms221810028

**Published:** 2021-09-17

**Authors:** Julia Doroszkiewicz, Magdalena Groblewska, Barbara Mroczko

**Affiliations:** 1 Department of Neurodegeneration Diagnostics, Medical University of Białystok, 15-269 Białystok, Poland; mroczko@umb.edu.pl; 2 Department of Biochemical Diagnostics, University Hospital in Białystok, 15-269 Białystok, Poland; magdalena.groblewska@umb.edu.pl; 3 Department of Biochemical Diagnostics, Medical University of Białystok, 15-269 Białystok, Poland

**Keywords:** gut microbiota, dysbiosis, gut–brain axis, central nervous system, enteric nervous system, neuroinflammation, neurodegeneration, neuropsychiatric disorders

## Abstract

The gut microbiome has attracted increasing attention from researchers in recent years. The microbiota can have a specific and complex cross-talk with the host, particularly with the central nervous system (CNS), creating the so-called “gut–brain axis”. Communication between the gut, intestinal microbiota, and the brain involves the secretion of various metabolites such as short-chain fatty acids (SCFAs), structural components of bacteria, and signaling molecules. Moreover, an imbalance in the gut microbiota composition modulates the immune system and function of tissue barriers such as the blood–brain barrier (BBB). Therefore, the aim of this literature review is to describe how the gut–brain interplay may contribute to the development of various neurological disorders, combining the fields of gastroenterology and neuroscience. We present recent findings concerning the effect of the altered microbiota on neurodegeneration and neuroinflammation, including Alzheimer’s and Parkinson’s diseases, as well as multiple sclerosis. Moreover, the impact of the pathological shift in the microbiome on selected neuropsychological disorders, i.e., major depressive disorders (MDD) and autism spectrum disorder (ASD), is also discussed. Future research on the effect of balanced gut microbiota composition on the gut–brain axis would help to identify new potential opportunities for therapeutic interventions in the presented diseases.

## 1. Introduction

When we think of the intestines, we associate them with rather basic and somewhat primitive functions that include the processing of food to deliver energy to the body. However, this type of understanding of the role of the gut appears to be inaccurate. The gut is innervated by the enteric nervous system (ENS), which acts independently from the central nervous system (CNS). However, there is a connection between these two parts of the human nervous system that allows for the exchange of information [1]. Knowledge of the gut function and its connection to the CNS through the ENS has prompted researchers to postulate the existence of a specific network called the “gut–brain axis”. The network is based on a complex system, including the vagus nerve, with both sympathetic and parasympathetic input, as well as certain gut hormones and cytokines [2,3].

The gut microbiota is a crucial part of the gastrointestinal tract. Recent data suggest that the connection between the gut and the brain should also be extended to the intestinal microbiome, creating the specific “microbiota–gut–brain axis”, as the latter role of bacterial flora within the gut has significantly emerged. Interestingly, it is postulated that microbes may participate in the development of the nervous system. This hypothesis has been confirmed in germ-free mouse models, which are considered the gold standard for microbiota studies. These studies have demonstrated impaired neurogenesis and morphology in the hippocampus and amygdala of the tested animals [4,5]. Furthermore, a different study conducted on germ-free mice revealed a disrupted microglia morphology and its processes, resulting in disturbed activation and response to pathogens [6]. These results have led to the establishment of a connection between pathological changes in the gut microbiota and neurological diseases. This review summarizes recent findings connecting the areas of microbiology, neurology, and the interplay between the gut microbiota and the brain in selected neurological diseases.

## 2. Gut Microbiota

The gut microbiota can be defined as a collection of microorganisms, primarily bacteria, which colonize the human gastrointestinal (GI) tract. The number of microorganisms inhabiting the GI tract has been estimated to range between 10^13^ and 10^14^, with an approximate weight of 2 kg, outnumbering the cells in the whole human body [7]. The normal human gut microbiota comprises two major anaerobic phyla, *Bacteroidetes* and *Firmicutes.* Interestingly, the ratio between these two species may be a relevant biomarker of gut dysbiosis in obesity [8]. Other identified types occur in small numbers and include some species from the *Proteobacteria*, *Cyanobacteria*, *Verrucomicrobia*, and *Actinobacteria* phyla [9]. In order to explain the influence of microbiota on the human body, we can divide the microorganisms that comprise the microbiota into “healthy” types, which exert a positive impact on the body, and “unhealthy” types, which may cause harm to the host. By way of illustration, it is believed that *Actinobacteria*, particularly *Bifidobacterium sp.*, demonstrate anti-inflammatory properties and exert a positive effect on the integrity of the intestinal barrier [10]. By contrast, *Clostridium difficile* is the leading cause of diarrhea [11]. The human gut microbiome contains around 4 million different genes, which is over 100 times more than the total human genes present in the human genome [12]. 

### 2.1. Changes in the Composition of the Intestinal Microbiome through Life 

It is believed that in normal conditions the fetus is germ-free, and the colonization and further development of the microbiota commences at birth. Interestingly, the method of delivery exerts a significant effect on the gut microbiota composition in newborns and infants. A recent study revealed that children born through caesarian section display diminished microbial diversity, primarily with decreased levels of *Bacteroides* or even their absence, which perseveres throughout the years. Moreover, after some time, it is only *Bacteroides* that differentiate the microbiota of babies delivered by C-section from the microbiota of those delivered vaginally [13]. Furthermore, fecal microbiota in vaginally delivered babies is broadly similar to that of their mothers, while in newborns delivered through caesarian section there is only 41% resemblance [14]. 

Another factor that significantly affects the gut microbiota is the feeding method. It has been demonstrated that breastfed infants show a higher level of dominance for *Bifidobacteria*, while formula-fed babies may display an increased prevalence of *Bacteroides* [15]. New studies reveal that environmental factors may cause modifications in the gut microbiota throughout life. Due to these factors, considerable changes occur in a child’s microbiota within the first 2–3 years of life. Following that, a certain degree of stabilization is achieved in the third year of life [16,17], after which time the stability is gradually lost over the years. Throughout life, the microbiota changes and diversifies, with a gradually increasing proportion of *Bacteroides* and *Clostridium.* Centenarians, in particular, exhibit greater abundance of *Enterobacteriaceae* [18,19].

### 2.2. Factors Influencing Gut Microbiota

Environmental factors such as diet, antibiotics, and infections may cause unhealthy diversification of the microbiota and may lead to increased intestinal permeability known as “leaky gut syndrome”. This might facilitate an improper and unnecessary immune response that enhances inflammation that is already occurring in the digestive tract [20]. As diet has a considerable impact on the gut microbiota composition, multiple studies have indicated that it is the most important modifying factor [21,22]. The Western diet, characterized by an increased dietary intake of saturated fat, sugar, and red meat, results in a shift to *Firmicutes*, with a decreased abundance of *Bacteroides* and a disturbed *Firmicutes/Bacteroidetes* ratio [23]. In contrast, vegetarian or Mediterranean diets, characterized by the high consumption of vegetables and fruit, healthy fats, and whole grains; moderate consumption of red wine; and low consumption of red meat, result in healthier, more diverse microbiota. These changes include an increase in *Bacteroides*, with a decrease in *Firmicutes* [24], which is considered a neuroprotective factor associated with improved cognitive function and a lower risk of Alzheimer’s disease (AD) [25,26,27].

Antibiotic consumption, and in particular antibiotic overuse, is one of the main factors causing dysbiosis. As the majority of prescribed antibiotics are broad-spectrum antibiotics, they eliminate both “good” and “bad” bacteria [28]. Furthermore, increased stress levels caused by modern lifestyles may also exert a negative effect on the gut microbiota and its diversity, and may result in an increased abundance of *Clostridium* [29]. However, there are factors that can improve the gut microbiota such as exercise, which enhances microbial diversity and promotes the presence of bacteria producing butyrate, known for its health-promoting and anti-inflammatory properties, as well as its capacity to increase insulin sensitivity [30,31,32]. Moreover, the consumption of polyphenols in green tea, red wine, and other dietary products can also promote the growth of healthy and helpful bacteria [33]. 

### 2.3. Functions of the Gut Microflora

The main functions of the gut microbiota are nutrient metabolism and the synthesis of vitamins. It also participates in the breakdown of drugs and other xenobiotics. During these processes, the microbiota releases a wide range of metabolites and small molecules that affect the body. The digestion of dietary fiber, a process that is possible only with the participation of gut microbes, results in the production of short-chain fatty acids (SCFAs); excretion of intestinal gases, such as methane and carbon dioxide; and the release of modest amounts of lactate and alcohols [34]. Importantly, SCFAs are not only a source of energy but they also affect the maturation of the microglia in the CNS. They may act as signaling molecules within the CNS [6,35]. It has also been demonstrated that enteric microbes have the ability to metabolize primary bile acids to secondary forms. These bile acids can also perform metabolic activities, including their signaling role within the nervous system [36]. 

Any alterations in the composition, diversity, or excessive functionality of the gut microbiota may constitute potential pathogenic factors. Importantly, normal gut microbiota can stimulate the immune system and induce the release of proinflammatory cytokines such as IL-1β, IL-6, or TNF-α [37]. As a result, the microbiota impacts the immune response, thus protecting the host from pathogens [8]. Moreover, continual changes in epithelial cells may serve as another protective mechanism within the gut that prevents enhanced inflammation in the intestines [37]. Proliferation of intestinal epithelial cells is a clearing mechanism that leads to the replacement of old cell layers and helps to isolate pathogens from body cavities. 

## 3. Gut–Brain Interplay 

### 3.1. Metabolites Produced by the Gut Microbiota

The connection between the intestines and the CNS involves many pathways mediated by various substances synthesized by microbes. The gut microbiota is capable of producing and releasing some active metabolites that may serve as neuromediators participating in communication with the CNS and affecting the brain. Short-chain fatty acids (SCFAs), aromatic amino acids, and bile acids are the main substances from the microflora affecting the brain. SCFAs consist mostly of acetate, butyrate, and propionate, which could be the products of bacterial fermentation of carbohydrates. Interactions between these acids and the gut may be mediated through binding to G-protein-coupled receptors [38]. 

### 3.2. Direct and Indirect Effects of Gut Microbiota on the CNS

**Figure 1:**
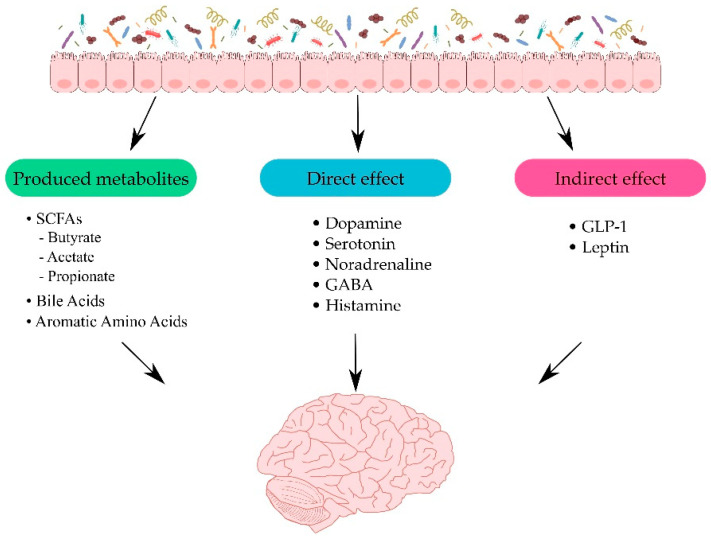
Pathways between the intestines and the central nervous system (CNS). Figure illustrates how the gut microbiota can interact with CNS through several pathways. Firstly, through produced metabolites such as short-chain fatty acids (SCFAs) [38]; secondly, directly with neurotransmitters for example dopamine and serotonin [39,40,41]; and lastly, indirectly influencing the release of enteric hormones [44,45,46].

Neurotransmitters and their precursors produced in the gut may also affect their levels in the brain. Besides being obtained from the breakdown of food, neurotransmitters can also be produced by bacteria. By way of illustration, *Escherichia coli* can release dopamine, serotonin, and noradrenaline, while *Lactobacilli* produce serotonin, GABA, acetylcholine, and histamine [39,40,41], which can influence the host brain. This mechanism has been proposed to play an important role in the development of certain neurological diseases, including Alzheimer’s disease, Parkinson’s disease (PD), depressive disorders, and autism spectrum disorders [42,43]. Furthermore, SCFAs are capable of indirectly affecting the gut–brain axis by inducing the release of some gut hormones, such as glucagon-like peptide-1 (GLP-1) and leptin, through enteroendocrine cells. These enteric hormones may interact with the vagus nerve and even brain receptors [44,45,46]. The effects of the gut microbiota on the brain are presented in Figure 1.

Interestingly, a number of studies indicate the contribution of SCFAs to the maintenance of physical barriers, such as the blood–brain barrier (BBB) or intestinal barrier by impacting the tight junctions between cells [46,47,48]. Similarly, bile acids can activate receptors in the host and act as signaling particles, and can affect barrier permeability [49,50]. Furthermore, lipopolysaccharide (LPS) produced by bacteria is able to influence BBB permeability by inducing the release of inflammatory cytokines [51].

### 3.3. Relevance of Healthy Gut Microbiota

Experiments conducted on germ-free mice have confirmed the importance of normal microbiota for many essential processes in the brain. One of them is the metabolism of serotonin, a neurotransmitter responsible for mood and appetite, which is mostly produced by enterochromaffin cells of the gastrointestinal tract [52]. Tryptophan is a precursor for this neuromediator. Recent studies have demonstrated that germ-free mice had increased tryptophan levels in plasma and decreased serotonin levels in the serum [53,54,55]. Moreover, the rodents exhibited a decreased expression of a crucial neurotrophic factor, brain-derived neurotrophic factor (BDNF), which is responsible for maintaining and promoting neurogenesis [56]. Furthermore, these animals also presented cognitive impairment, problems with sociability, and depressive and anxious behaviors [55]. 

Although germ-free mouse models are the gold standard for gut microbiome research, antibiotic-treated models constitute a more accessible and less expensive approach. Antibiotics may be used to modify the gut bacteria, analyze changes in the microbiota, and evaluate their impact on the brain [57]. It has been demonstrated that experimental treatment with oral antibiotics not only induces intestinal dysbacteriosis, but also leads to an imbalance in the gut–brain axis. Moreover, antibiotic-induced changes in microbial composition also cause certain neurobehavioral alterations, such as increased anxiety and “depressive-like” behaviors, as well as neuronal activation in different brain regions of mice [58].

## 4. Gut and Neurological Diseases

Recent data suggest that neuroinflammation may be a pathogenic factor in several neurodegenerative disorders. In a neuroinflammatory state, activation of the microglia and the release of proinflammatory proteins, such as TNF-α, IL-6, or MCP-1, as well as reactive oxygen species by glial cells and resident macrophages, might result in chronic neuroinflammation [20,59]. As the gut microbiota is believed to contribute to various pathogenic pathways, there are a growing number of studies linking changes in the healthy microbiome to the development of a number of neurological diseases, including those with neurodegenerative etiology, as well as to certain neuropsychiatric disorders [3,60,61]. Likewise, diminished microbiota diversity throughout life may be connected with neurodegeneration [62].

**Figure 2:**
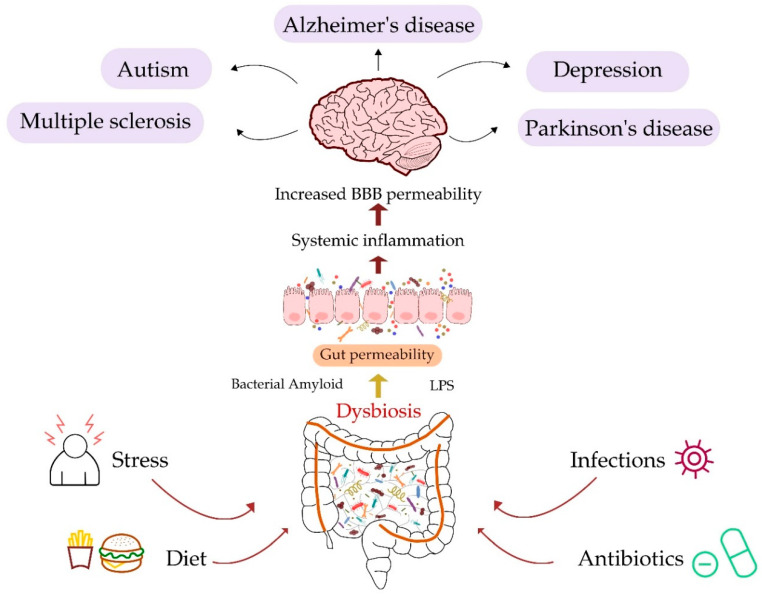
Schematic description of disturbances in the intestinal microbiota and diseases of the CNS. The figure depicts major factors, such as stress [29], diet [21], infections, and antibiotic intake [28], which can promote dysbiosis in the gut microbiota. Changes in the composition of the microbiome lead to gut permeability, influencing systemic inflammation and, as a result, may induce diseases of the CNS.

Although loss of intestinal epithelial cell integrity and chronic inflammation seem to be the main consequences of changes in the gut microbiota, neuroinflammation as well as neurodegenerative and neuropsychiatric disorders may also be the result of these changes. The importance of a healthy gut microbiota and its diverse composition for normal brain function has been confirmed in studies on rodent models [63]. It has been demonstrated that the presence of *Lactobacillus* bacteria exerts a positive effect on the brains of rats with experimental cerebral ischemia reperfusion injury via the inhibition of neural cell apoptosis and the reduction of oxidative stress through the downregulation of the TLR4/NF-kB signaling pathway [63]. As described in previous sections, a number of factors, including diet, stress, infections, and antibiotic use, may result in gut microbiota dysbiosis. The connection between changes in the gut and CNS disorders is shown in Figure 2, with further description in later sections.

### 4.1. Alzheimer’s Disease

Alzheimer’s disease is the most prevalent neurodegenerative disorder. AD is a progressive disease whose first clinical symptoms appear decades after the onset of pathological changes in the brain, thus making older individuals the most affected age group. Hallmarks of the disease include progressive accumulation of amyloid-beta (Aβ) plaques, formed following the cleavage of amyloid precursor protein (APP), in the brain and neurofibrillary tangles (NFTs), which consist of hyperphosphorylated tau protein [64]. 

The etiology of AD has not been fully elucidated, but a number of factors influence the pathogenesis of the disease. Proposed hypotheses on the origin of AD includes gradual accumulation of Aβ in the AD brain, followed by progressive deposition of the Tau protein. Another hypothesis suggests the role of soluble oligomers of Aβ and/or Tau protein as the most harmful factors affecting the brain tissue [65,66]. Moreover, the contribution of the immune system to AD pathogenesis has also been suggested. Insoluble deposits of Aβ may be recognized by the immune system as a foreign material, triggering the inflammatory cascade, which leads to neuronal damage. Amyloid plaques and NFTs generate inflammation within the brain, primarily throughout activation of the microglia and astrocytes.

The neuroinflammation hypothesis in the pathogenesis of AD is associated with certain gut microbiota alterations. Neuroinflammatory processes occur with ongoing systemic inflammation, which may further intensify neuroinflammation [20,67,68]. Furthermore, it has recently been suggested that AD is initiated is the gut, and not the brain, from where it subsequently proceeds to the brain. The hypothesis was confirmed in a study by Sun et al., in which Aβ_1–42_ oligomers were injected into the wall of the gastrointestinal tract of mice. Initially, the oligomers of Aβ_1–42_ were internalized into enteric cholinergic neurons, but after one year of observation, relocated amyloid was found in the brain of rodents, which exhibited gastrointestinal tract dysfunction and cognitive deficits [69]. The findings confirmed that intra-gastrointestinal oligomers of Aβ_1–42_ could disturb not only enteric function, but also induce AD in the studied animal model of AD. Moreover, as Aβ from the periphery could contribute to the Aβ burden in the brain, these results may support the hypothesis that Aβ has prion-like properties [70]. Furthermore, the discovery of amyloid migration may prove the connection between the gut and neuroinflammation in AD. 

Recent data also link changes in the gut microbiota with AD [71]. Changes in the phyla *Bacteroidetes* and *Firmicutes*, unrelated to age, have been demonstrated in mouse models of AD [72,73,74]. The results from animal models have also been confirmed in human studies using gene sequencing techniques. Human fecal samples showed similar results, with alterations in the microbiome, including decreased *Firmicutes* and *Bifidobacterium*, but increased *Bacteroidetes* in AD patients [75]. Another study conducted among cognitively impaired patients with brain amyloidosis revealed an increased abundance of *Escherichia/Shigella*, known as pro-inflammatory bacteria, with a simultaneous decrease in anti-inflammatory *Eubacterium rectale* in comparison with healthy controls and individuals without amyloidosis in PET imaging. Additionally, in these patients, a positive correlation was found between increased blood levels of pro-inflammatory cytokines, such as IL-1β and CXCL2, and a component of the inflammasome complex NLR family pyrin domain containing 3 (NLRP3), and abundance of *Escherichia/Shigella* [75,76]. When the CSF levels of various AD biomarkers were investigated in a study by Vogt et al., a significant relationship was revealed between YKL-40 concentrations, and an increased abundance of *Bacteroides* and decreased presence of *Turicibacter* [75]. 

The connection between the gut and the brain in AD has also been confirmed in studies exploring the transfer of healthy microbiota from wild type mice to mouse models of AD. Normalization of the gut microbiome led to the reduced formation of Aβ plaques and neurofibrillary tangles, decreased glial reactivity, and improved cognitive performance [77]. Moreover, the results of the study are in line with human studies showing that transplantation of healthy microbiota to AD patients suffering from *Clostridium difficile* infection resulted in improved cognitive function, as demonstrated by the Mini-Mental State Exam (MMSE) score [78,79]. On the other hand, it was revealed that pathological changes in microbiota composition, e.g., a decrease in *Bifidobacteria* and the overgrowth of *Clostridium difficile*, may stimulate a shift in the expression of proinflammatory molecules [3,18]. 

Interestingly, a specific kind of amyloid has also been identified on the bacterial cell surface. The first discoveries of this protein were described in *Escherichia coli* curli, the proteinaceous components of bacterial extracellular fibers [80]. Similar abilities to produce bacterial amyloids have been discovered in other species, such as *Staphylococcus*, *Streptococcus*, *Salmonella*, and *Klebsiella*. Bacterial amyloid plays an important role in the building of a microbial biofilm, which prevents gut bacteria eradication [81]. Although the primary structures of bacterial and CNS amyloids are not similar, their tertiary structures reveal a significant similarity [82]. As a result, the presence of bacterial amyloid in the gut is thought to affect the immune system, consequently intensifying reaction to the formation of neuronal amyloids [81]. Knowing that bacterial amyloids can cross physiological barriers, it has been suggested that they contribute to the development of AD [83]. 

Lipopolysaccharide (LPS), which is known for its pro-inflammatory properties, is present in the membrane of Gram-negative bacteria such as *Bacteroides*. It has been proven that LPS is capable of generating inflammation and may mediate the release of many proinflammatory cytokines through Toll-like receptor-4 (TLR-4) [84]. This receptor contributes to the activation of the microglia at the earliest stages of Aβ deposition in the brain, which has been demonstrated in mouse models of AD with TLR-4 deletion, showing enhanced amyloidosis [85]. 

Another rodent study on bacterial LPS revealed that following the intraperitoneal injection of LPS, mice showed elevated hippocampal levels of Aβ_1-42_ with simultaneous cognitive defects [86]. The importance of this bacterial endotoxin for amyloid fibril formation has been confirmed by in vitro experiments that demonstrated that LPS from *Escherichia coli* could potentiate Aβ organization in compact fibrils. These results confirm that LPS is a key factor in the kinetics of Aβ fibrillogenesis [87]. Furthermore, a significant increase in LPS levels has been observed in the brain samples obtained from AD patients that were co-localized with Aβ plaques, suggesting that this bacterial molecule has the ability of to pass through physiological barriers into the brain [88,89]. Consequently, elevated LPS levels have been found in the plasma of AD patients [90]. This finding is consistent with the aforementioned hypothesis of the “leaky gut syndrome” and loss of integrity of intestinal and BBB barriers with age, which contribute to neuroinflammation. Furthermore, knowing that *Bifidobacterium* and *Lactobacillus* exert a beneficial effect on LPS levels and barrier integrity, a decrease in the abundance of these bacteria may play a significant role in the development of AD [91].

### 4.2. Parkinson’s Disease

Parkinson’s disease is the second most common neurodegenerative disorder [92], which may also be linked to disturbances in the brain–gut–microbiota axis. PD is a chronic, progressive disease characterized by both motor and non-motor features. It mostly occurs in men and women over 40 years of age and its incidence increases with age [93,94]. Tremor, bradykinesia, and postural instability are the primary motor symptoms, while cognitive decline, sleep disturbances, depression, and anxiety are the main non-motor symptoms [95]. The causes of PD include deterioration of the dopaminergic neurons in the extrapyramidal tract of the midbrain, which is believed to be responsible for motor dysfunction [96]. The histopathological hallmark of PD is the presence of misfolded, insoluble α-synuclein, which may aggregate into Lewy bodies in neurons, thus inducing neurodegeneration [97]. Similarly to AD, neuroinflammation also plays a role in the pathophysiology of PD, which is evident in microgliosis and astrogliosis [98]. 

Non-motor symptoms attributable to the digestive system are particularly common in patients with PD. Hypersalivation, which is the result of impaired swallowing, and constipation, caused by motility changes, are the most common dysfunctions of the gastrointestinal tract [99]. These observations support the hypothesis that PD may originate within the gut. Moreover, constipation may be linked to enteric nervous system degeneration caused by the aggregation of alpha-synuclein, increased intestinal permeability, and local inflammation [100]. It has also been observed that there are strong associations between changes in intestinal motility, which may predate neurological manifestations of the disease by a number of years, a well as a subsequent diagnosis of PD. It has been demonstrated that constipation significantly increases the risk of developing PD [101,102]. 

It has been suggested that disturbed gut microbiota, which is responsible for intestinal motility, increased permeability, and chronic local inflammation, could be considered an important factor in the pathophysiology of PD. As dysfunction of the gastrointestinal system is a characteristic feature of PD, the gut microbiota composition in PD patients has also been investigated. It has been shown that patients suffering from PD have an increased abundance of *Enterobacteriaceae*, which was positively correlated with postural instability [103]. In contrast, another study demonstrated that certain families of healthy bacteria, such as *Prevotellaceae* and *Lachnospiraceae*, were reduced in the fecal samples in PD patients [104]. *Prevotellaceae* are known for participating in the production of mucins, which play a crucial role in maintaining intestinal permeability. Thus, their reduced presence might be linked to the “leaky gut syndrome” [105]. *Lachnospiraceae*, such as *Blautia*, *Coprococcus*, and *Roseburia*, participate in the production of SCFAs which are known for their anti-inflammatory and gut protective properties, which may also contribute to altered permeability [104,106]. 

Interestingly, studies on animal models of PD have demonstrated a connection between motor deficits, neuroinflammation, and the gut microbiota. Mouse models of PD have shown a disturbed gut microbiota, with a decreased presence of *Firmicutes* and *Clostridiales* and an increased abundance of *Proteobacteria* and *Enterobacteriales*. Experimental transplantation of fecal microbiota not only ameliorated microbiota composition and reduced SCFAs concentration in mouse feces, but also diminished the activation of microglia and astrocytes within their brains [107]. 

A recent study demonstrated that providing germ-free mice with selected microbial metabolites resulted in neuroinflammation and detectable PD-like physical symptoms [108]. Consequently, transplantation of the microbiota from healthy individuals to mice overexpressing α-synuclein resulted in diminished motor impairments in comparison with the animals treated with microbiota from PD patients [108]. Additionally, a recent study conducted on aged rats with aggregated α-synuclein in the intestinal submucosal plexus, which were exposed to transgenic *E. coli* that produced bacterial amyloids, displayed a significant reaction to bacterial curli. It has been demonstrated that the production of α-synuclein within the intestines and its accumulation in the brain of examined rodents is intensified, which further prompts microgliosis and astrogliosis. Moreover, the rat brains showed an increased expression of TLR-2, TNF-α, and IL-6, which may indicate that amyloids produced by bacteria provoke α-synuclein aggregation and, as a result, an innate immune system response [109,110]. Another microbial metabolite, LPS, might be a crucial factor in the pathophysiology of PD, similarly to AD. LPS-induced inflammation in rodent models of PD activate the microglia, which results in dopaminergic neuron damage and loss [99,111]. 

It is worth mentioning that a large number of PD patients are infected with *Helicobacter pylori.* For a number of years, the presence of gastric ulcers, which are triggered by *H. pylori*, has been linked to PD [112]. On the other hand, this bacteria is also known for impairing the absorption of levodopa, a key drug in the treatment of motor aspects of PD [113]. It has been demonstrated that comorbidity between PD and *H. pylori* infection is related to more severe manifestations of PD and more significantly impaired motor function [114]. Interestingly, the existence of *H. pylori* infection might also increase the incidence of PD [112,115]. 

### 4.3. Multiple Sclerosis

In contrast with AD and PD, multiple sclerosis (MS) is a disease affecting mostly young adults, particularly women [116]. It is a demyelinating CNS disease with an inflammatory component. MS is characterized by chronic inflammation both in white and grey matter of the brain and the spinal cord, which causes destruction of the myelin that covers the neurons [117]. Although the mechanisms underlying MS are not fully understood, it is postulated that malfunction of the immune system is the most probable cause of the disease [118]. Apart from chronic inflammation, altered selectivity of BBB in MS brain has been observed. This state facilitates the migration of immune cells (mostly T-cells) to the nervous system and penetration of the brain. Following infiltration into the CNS, T-cells start to recognize myelin as a trigger to the immune system, which causes enhanced inflammation and results in demyelination [117,119].

The pathophysiology of MS may be also linked to genetic and environmental factors [120]. Obesity in early life [121], decreased vitamin D levels in the blood, and insufficient exposure to sunlight [122], as well as smoking [123], are the established and most frequently described causes. All of these aspects can also indirectly affect the gut microbiota. As microbes may control immunity by regulating T-cells, the gut microbiota has received attention as an important factor in MS pathology. 

It has been demonstrated that there are considerable differences between stool samples from MS patients and those from healthy controls. MS samples revealed decreased levels of *Bacteroidetes, Clostridium*, *Fecalibacterium*, and *Prevotella* taxa (the last one produces propionate, which is a SCFA) [124,125,126]. Moreover, increased abundance of *Methanobrevibacter* and *Akkermansia muciniphila* has been observed in different types of MS [127]. In addition, transplantation of the microbiota from MS patients to germ-free mice resulted in intensified experimental autoimmune encephalomyelitis (EAE), an animal type of demyelinating diseases, in contrast with germ-free mice treated with healthy microbiota [128]. These results suggest the potential involvement of the microbiota in the development of MS and its impact on disease progression. 

It has been revealed that various metabolites produced by commensal *Clostridium*, such as SCFA butyrate, propionate, and acetate, may differentially induce T-regulatory cells, affecting the balance between pro- and anti-inflammatory cells in MS patients [129]. Moreover, studies among MS patients treated with vitamin D have revealed an altered composition of the gut microbiota, with elevated levels of *Faecalibacterium*, an anti-inflammatory bacteria that produces butyrate [125]. These results may also confirm the positive effect of vitamin D supplementation for MS patients. 

### 4.4. Major Depressive Disorder

Major depressive disorder (MDD) is a mental disorder and is one of the leading causes of disability, morbidity, and mortality in developed countries. In 2017, more than 264 million people worldwide were affected by MDD [130]. The main symptoms of MDD include low mood, difficulties in concentration, fatigue, appetite alteration, and digestive and sleeping problems. For an appropriate diagnosis, the symptoms must be present continuously for a minimum of a 2-week period [131]. The pathophysiology of MDD is still not fully understood. However, it is suggested that deficiency in monoamine neurotransmitters, such as serotonin, noradrenaline, and dopamine, may be the key cause of the disorder [132]. Another cause of the disease may be systemic inflammation, with elevated blood cytokine levels, which also demonstrates that depression is a systemic disease. Systemic inflammation also leads to neuroinflammation and the activation of microglia and astrocytes, contributing to the development of MDD, affecting behavior and emotions [133,134,135,136]. 

As previously described, the gut and the brain communicate in a bidirectional manner and interactions between these organs are important for the development of the CNS. Moreover, the gut microbiota exerts a significant impact on the CNS and may act as a mediator in communication between the gut and the brain. Studies on germ-free mice show that changes in the gut microbes may promote anxiety-like behaviors in these animals, whose effects persist after colonization with normal intestinal microbiota [137]. Interactions between the gut and the brain are essential to the development of stress systems within the CNS, with a possible critical time window, after which reconstitution of the microbiota may not be able to normalize the behavioral phenotype. Another study in which the microbiota from depressed patients was transferred to rats, revealed enhanced depression-like behaviors in these animals, with disturbed tryptophan metabolism [138].

The existing body of knowledge of the gut microbiota and microbiota-released molecules have prompted researchers to consider disturbances in the gut–brain axis as a new aspect of MDD pathology. Some studies have revealed that gut microbiota composition in depressed patients was significantly altered in comparison with healthy controls. MDD patients had an increased abundance of *Bacteroidetes* and *Proteobacteria*, with a decrease in *Firmicutes*, *Bifidobacterium*, and *Lactobacillus* [139,140,141]. Similar results have been obtained from animal studies, with an enhanced proportion of *Bacteroidetes* and a decreased proportion of *Firmicutes* in various depression models [142,143,144]. 

Moreover, it has been identified that the presence of *Coprococcus*, a bacterial species that produces a beneficial SCFA, butyrate, in the patient’s gut, is connected with indicators of a higher quality of life, such as perceived health status, physical functioning, vitality, emotional well-being, and social functioning. Interestingly, *Coprococcus* was found to be diminished in MDD patients [145]. A recent meta-analysis demonstrated a decline in depression scores in MDD patients after restoration of the microbiota with probiotics, which also confirms essential connections between depression and the gut microbiota [146].

### 4.5. Autism Spectrum Disorder 

Autism spectrum disorder (ASD) is a neurodevelopmental disorder characterized by impairments in communication and social interactions, repetitive and stereotyped behaviors, and restricted interests [147]. It is commonly diagnosed in infants, mostly boys, between 1 and 2 years of age [148]. However, as ASD consists of a broad range of conditions, it may be diagnosed later in life, also in adults [149]. Although the exact cause of ASD is not fully understood and is highly complicated, it is linked to genetic and environmental input [150].

Apart from psychological aspects, ASD patients exhibit gastrointestinal symptoms, such as diarrhea and/or constipation, as well as abdominal pain [151]. Recognizing the relationship between the gut and the brain, researchers started investigating the microbiota of ASD patients. It has been demonstrated that people suffering from ASD show an increased abundance of *Clostridium* and *Lactobacillus* species [152,153]. Furthermore, *Bacteroidetes* dominate in the intestines, with a concurrent decrease in *Firmicutes* [154]. Another study revealed decreased levels of beneficial species *Prevotella* and *Coprococcus* in the intestinal microflora of autistic children in comparison with healthy controls [155]. Similar results have been obtained in animal studies. It has been demonstrated that the valproic acid rat model of autism exhibits changes in the diversity and number of species, and has a composition of gut bacteria similar to those observed in human autism [156]. Moreover, significantly increased serum levels of bacterial LPS, as well as IL-1beta and IL-6, the biomarkers of inflammation, have been observed in adult autistic patients in comparison with healthy controls. Furthermore, these results were inversely correlated with socialization scores [157]. These findings confirm the role of microbiota in ASD. However, the significance of low-grade endotoxemia in the pathophysiology of autism needs further investigation. 

Importantly, it seems that exposure in utero to inflammation resulting from disturbances in the maternal gut microbiota may increase the probability of ASD occurrence in children. This has been confirmed in studies on mouse models, which indicate that changes in the maternal gut microbiome promote neurodevelopmental abnormalities in mouse offspring [158]. 

## 5. Conclusions

In recent years, the gut microbiota and its importance for the functioning of the human body has generated considerable interest among researchers, although we still do not know whether alerations in the microbiota trigger pathological changes or coexist with them. However, a balanced composition of the gut microbiota and the production of various bacterial metabolites have proven their profound significance for host health, including for the CNS. The gut–brain axis, which may be defined as a complex interplay between the function of the gastrointestinal system, including the enteric nervous system, the activity of our intestinal microbes, and the CNS, may influence the development of various brain diseases. 

The body homeostasis may be affected by the pathological shift in the microbiome and its altered metabolism, thus promoting the development of different neurological and neuropsychiatric disorders. These gut microbiome-related diseases of the CNS include neurodegenerative disorders, such as Alzheimer’s disease, Parkinson’s disease, and multiple sclerosis, as well as depression and autism spectrum disorders. Therefore, the intestinal microflora can be seen as an important factor in the development of CNS and the progression of various neurological diseases. 

Although the gold standard in microbiota analysis is a fecal material examination, a multiplatform analysis of its metabolites may also provide valuable information regarding the state of the microbiome. Moreover, the gut microbiome could be a potential new target for the treatment of these diseases. Prevention of gut dysbiosis by probiotics may also provide protection from the disorders described above. A summary of the microbiota changes observed in neurological diseases is presented in Table 1.

## Figures and Tables

**Table 1:** Changes in the microbiota in neurological diseases.

Type of Disease	Bacteria	Direction of Changes	Author
Alzheimer’s Disease	*Bacteroidetes*	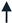	[75,76]
	*Escherichia/Shigella*	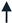	[76]
	*Firmicutes*	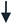	[75]
	*Eubacterium rectale*	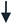	[76]
Parkinson’s Disease	*Enterobacteriaceae*	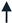	[103]
	*Helicobacter pylori*	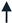	[114]
	*Prevotellaceae*	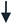	[104]
	*Lachnospiraceae*	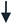	[104]
Multiple Sclerosis	*Methanobrevibacter*	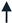	[127]
	*Akkermansia muciniphila*	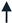	[127]
	*Bacteroidetes*	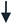	[124]
	*Clostridium*	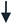	[124]
	*Fecalibacterium*	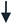	[125]
	*Prevotella*	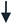	[126]
Major Depressive Disorder	*Bacteroidetes*	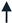	[138]
	*Proteobacteria*	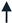	[138]
	*Firmicutes*	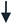	[138]
	*Bifidobacterium*	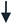	[140]
	*Lactobacillus*	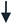	[140]
	*Coprococcus*	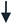	[144]
Autism Spectrum Disorder	*Bacteroidetes*	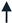	[154]
	*Clostridium*	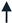	[153]
	*Lactobacillus*	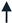	[153]
	*Firmicutes*	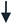	[154]
	*Prevotella*	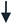	[155]
	*Coprococcus*	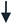	[155]

## Data Availability

Not applicable.

## Abbreviations

AD	Alzheimer’s disease
ASD	Autism spectrum disorder
Aβ	Amyloid beta
BDNF	Brain-derived neutrophic factor
CNS	Central nervous system
ENS	Enteric nervous system
GLP-1	Glucagon-like peptide
LPS	Lipopolysaccharide
MDD	Major depressive disorder
MS	Multiple sclerosis
NFTs	Neurofibrillary tangles
NLRP3	NLR family pyrin domain containing 3
PD	Parkinson’s disease
SCFA	Short-chain fatty acid
TLR-4	Toll-like receptor-4
